# Antioxidant Treatment Promotes Prostate Epithelial Proliferation in *Nkx3.1* Mutant Mice

**DOI:** 10.1371/journal.pone.0046792

**Published:** 2012-10-15

**Authors:** Erin E. Martinez, Philip D. Anderson, Monica Logan, Sarki A. Abdulkadir

**Affiliations:** 1 Department of Pathology, Microbiology, and Immunology, Vanderbilt University Medical Center, Nashville, Tennessee, United States of America; 2 Department of Biochemistry and Cancer Biology, Meharry Medical College, Nashville, Tennessee, United States of America; 3 Department of Cancer Biology, Vanderbilt University Medical Center, Nashville, Tennessee, United States of America; The University of Texas M. D. Anderson Cancer Center, United States of America

## Abstract

Discordant results in preclinical and clinical trials have raised questions over the effectiveness of antioxidants in prostate cancer chemoprevention. Results from the large-scale Selenium and Vitamin E Cancer Prevention Trial (SELECT) showed that antioxidants failed to prevent, and in some cases promoted, prostate cancer formation in men without a history of the disease. One possible explanation for these alarming results is the notion that the effects of antioxidant treatment on the prostate are modified by specific, intrinsic genetic risk factors, causing some men to respond negatively to antioxidant treatment. Loss of expression of the homeobox transcription factor NKX3.1 in the prostate is frequently associated with human prostate cancer. *Nkx3.1* mutant mice display prostatic hyperplasia and dysplasia and are used as a model of the early stages of prostate cancer initiation. While the mechanisms by which Nkx3.1 loss promotes prostate tumorigenicity are not completely understood, published data have suggested that elevated reactive oxygen species (ROS) associated with Nkx3.1 loss may be a causative factor. Here we have tested this hypothesis by treating Nkx3.1 mutant mice with the antioxidant N-acetylcysteine (NAC) for 13 weeks post-weaning. Surprisingly, while NAC treatment decreased ROS levels in *Nkx3.1* mutant mouse prostates, it failed to reduce prostatic epithelial hyperplasia/dysplasia. Rather, NAC treatment increased epithelial cell proliferation and promoted the expression of a pro-proliferative gene signature. These results show that ROS do not promote proliferation in the *Nkx3.1*-null prostate, but instead inhibit proliferation, suggesting that antioxidant treatment may encourage prostate epithelial cell proliferation early in prostate tumorigenesis. Our findings provide new insight that may help explain the increased prostate cancer risk observed with vitamin E treatment in the SELECT trial and emphasize the need for preclinical studies using accurate models of cancer.

## Introduction

Due to the high prevalence and significant treatment-related morbidity associated with human prostate cancer, there is a strong interest in preventive approaches. In order to accomplish this, a more thorough understanding of the relationship between oxidative stress and the steps of prostate tumor progression is needed. In recent years, extensive research has been devoted to the relationship between oxidative stress and the etiology of prostate cancer [1], [2], [3], [4]. In addition, the prostate gland has been associated with chronic inflammation [5], a condition linked to elevated oxidative stress. Many studies have proposed a positive correlation between elevated oxidative stress and prostate cancer progression and have argued the value of antioxidants in preventing prostate cancer (reviewed in [6]). However, it is notable that the majority, if not all, of these studies have employed models of late stage, aggressive disease, focusing on later steps in carcinogenesis rather than prevention of prostate cancer initiation [7], [8], [9], [10], [11], [12], [13], [14].

The Selenium and Vitamin E Cancer Prevention Trial (SELECT) was initiated in 2001 to conduct a large, randomized controlled clinical trial on the efficacy of the antioxidants selenium and vitamin E in the prevention of prostate cancer [15]. Results from two previously published clinical trials [16], [17] suggested that these two antioxidants could prevent prostate cancer development. However, initial results published in 2009 [18] showed that neither selenium, vitamin E, nor their combination significantly prevented prostate cancer in the study population. Follow-up results published in late 2011 [19] showed that vitamin E treatment increased rather than decreased the risk of development of prostate cancer. This concerning finding highlights the importance of understanding the role of ROS in prostate tumorigenesis. In fact, one of the lead authors of the SELECT trial has suggested that any success in future chemoprevention may reside in the identification of specific risk factors in individuals that will help determine the effect any agent may have on their tumor development [20].

NKX3.1 is a homeodomain transcription factor whose loss of expression correlates with human prostate cancer progression [21], [22], [23]. NKX3.1 expression is lost early in tumorigenesis, suggesting that it is an early step in the progression to malignant disease. While several studies have investigated the role Nkx3.1 loss plays in prostate cancer [24], [25], [26], [27], [28], [29], [30], [31], [32], [33], [34], much remains unknown. *Nkx3.1^−/−^* mice are a model of the early stages of prostate tumorigenesis, exhibiting hyperplasia and dysplasia at 8 weeks of age and progressing to prostatic intraepithelial neoplasia (PIN), a precursor lesion to prostate cancer, later in life [35], [36], [37]. With additional genetic lesions, such as the loss of one allele of the Pten tumor suppressor gene [38], these mice develop prostate cancer. Ouyang *et al.* showed that prostates of *Nkx3.1^−/−^* mice show dysregulation of several antioxidant and pro-oxidant control enzymes, accompanied by elevated oxidative stress [39]. They and others have suggested that increased oxidative stress may be an important way in which Nkx3.1 loss promotes prostate tumor initiation [40], [41]. However, the ability of oxidative stress to mediate the hyperplasia of the *Nkx3.1^−/−^* mouse prostate has not been examined.

In this study, we tested the ability of antioxidant treatment to prevent the prostate pathology of *Nkx3.1^−/−^* mice. Interestingly, we found that antioxidant treatment did not inhibit, but instead promoted, the hyperplastic phenotype of the *Nkx3.1^−/−^* prostate. NAC treatment of *Nkx3.1^−/−^* prostate also induced expression of a pro-proliferative gene signature, as demonstrated by Genome Set Enrichment Analysis (GSEA). This suggests that ROS restrain the proliferative potential of the prostate epithelium in the setting of Nkx3.1-loss. Our studies give new insight into the failure of antioxidants to prevent prostate cancer in healthy men.

## Materials and Methods

### Animals

*Nkx3.1^−/−^* mice have been described [36]. Mice were maintained at Vanderbilt University Medical Center in compliance with national and institutional animal welfare standards. For NAC treatment, *Nkx3.1^+/+^* and *Nkx3.1^−/−^* pups were weaned at 3 weeks of age and littermates were divided between NAC treatment cages or vehicle cages. Mice received vehicle or 5 mM NAC (Sigma) in drinking water *ad lib* beginning at weaning for 13 weeks. The pH of NAC solution was adjusted to that of regular drinking water. Analysis of water intake and weight data after the conclusion of the experiment showed that the NAC dosage achieved was 158.5 mg/kg/day in *Nkx3.1^+/+^* mice and 140.7 mg/kg/day in *Nkx3.1^−/−^* mice. At the end of 13 weeks of treatment, the mice were euthanized following BrdU intraperitoneal injection (50mg/kg) for prostate histological analysis. Animal protocol M/08/047 was approved by Vanderbilt's Institutional Animal Care and Use Committee.

### Quantitative reverse transcription-PCR (qRT-PCR)

Total RNA was extracted from snap-frozen mouse anterior prostate tissue according to the Trizol® manufacturer's protocol. RNA was treated with RQ1 Rnase-free DNAse (Promega) according to manufacturer's protocol and incubated at 37°C for 20 minutes, followed by purification using the RNA Clean Up protocol from the RNeasy Mini Kit (Qiagen). 1 ug RNA was subjected to reverse transcription using M-MLV Reverse Transcriptase (Invitrogen). Quantitative real time PCR was performed using SYBR® Green and the Applied Biosystems 7300 Real Time PCR system with gene-specific primers designed using Applied Biosystems Primer Express® software. The following primers were used: *18s* forward (5′-CGCCGCTAGAGGTGAAATTCT-3′), *18s* reverse (5′-CGAACCTCCGACTTTCGTTCT-3′), *Gpx2* forward (5′-TGACCCGTTCTCCCTCATG-3′), *Gpx2* reverse (5′-GCGCACGGGACTCCATAT-3′), *Prdx6* forward (5′-TCTGGCAAAAAATACCTCCGTTA-3′), *Prdx6* reverse (5′-GCCCCAATTTCCGCAAAG-3′), *Qsox1* forward (5′-GGCTGGGAGGGTGACAGTT-3′), and *Qsox1* reverse (5′-std 18 GCCCCTACCACCAAGCAA-3′), The expression of each mRNA was normalized to *18s* rRNA expression.

### ChIP-qPCR of Nkx3.1 binding sites in LNCaP cells

Chromatin immunoprecipitation (ChIP) was performed using the ChIP Assay kit (Millipore) as described by the manufacturer with the following modifications. LNCaP cells (ATCC) were grown in RPMI medium supplemented with 10% fetal bovine serum (FBS) and 1 nM dihydrotestosterone (DHT) for 48 hours. Cells were fixed in 1% formaldehyde at 37°C for 10 minutes to crosslink protein-DNA complexes. Next, cells were thoroughly washed with ice-cold PBS, pelleted, and resuspended in SDS lysis buffer [1% SDS, 10 mM EDTA, 50 mM Tris at pH 8.1]. Chromatin was sheared to a size of ∼300–500 base pairs and diluted 1∶10 with ChIP dilution buffer. An aliquot of the diluted sample (1%) was saved as input. Samples were precleared and precipitated overnight at 4°C with anti-NKX3.1 (Santa Cruz Biotechnology) or normal goat IgG (Santa Cruz Biotechnology) antibodies. Antibody complexes were collected with Protein A Agarose/Salmon Sperm DNA (Millipore) for 2 h and washed extensively per manufacturer's instructions. Samples were reverse cross-linked at 65°C overnight with 0.3 M NaCl and 30 µg of RNase A (Qiagen). Input and bound DNA were purified with a PCR Purification kit (Qiagen) and analyzed by qPCR (Applied Biosystems 7300) using SYBR Green. The following primers were used for qPCR: *QSOX1* forward (5′-CCTTCATTGCTATTCACTGGCTAA-3′), *QSOX1* reverse (5′-TCCCCAACTGCAATGCAAA-3′), *PRDX6* forward (5′- GGTGGCCGAAAGACTTTTTG-3′), *PRDX6* reverse (5′- TGGCTCTTCCTAAAGCTGTTATCA-3′), *GPX2* forward (5′- GAATCAGTCTAGCAAAGGATCAAACA-3′), and *GPX2* reverse (5′-GCATAGAGGGTGTAGTTACTGAGAACA-3′). Immunoprecipitated DNA was normalized to 1% input. Results are presented as mean ± SD.

### DHE staining

DHE staining was performed on anterior prostate tissue frozen in Tissue Tek® OCT embedding medium. 10 µm sections were cut and stained with 10 µM dihydroethidium (Molecular Probes) for 30 minutes in a 5% CO_2_ incubator and visualized on a Zeiss fluroescent microscope. Fluorescence intensity of each image was scanned and scored using Bio Rad GS-700 Imaging Densitometer and BioRad Quantity One® software.

### Histology and immunohistochemistry

Tissue was fixed overnight in 10% formalin solution and washed in 70% ethanol. Tissue processing and hematoxalin and eosin (H&E) staining was performed by the Vanderbilt Translational Pathology Shared Resource. For immunohistochemistry, paraffin embedded sections were deparaffinized, rehydrated, and steam/pressure antigen retrieval was performed. The following antibodies were used: anti-BrdU (mouse, 1∶200, Santa Cruz Biotechnology), anti-phospho histone H3 (rabbit, 1∶500, Millipore), anti-cleaved caspase-3 (rabbit, 1∶200, Cell Signaling), anti-smooth muscle actin (mouse, 1∶2000, Sigma), anti-p63 (PIN cocktail, Biocare Medical), anti-AR (rabbit, 1∶600 Santa Cruz Biotechnology), anti-p16 (rabbit, 1∶1000, Santa Cruz Biotechnology), anti-8-Hydroxydeoxyguanosine (8-OHdG) (mouse, 1∶1000, QED Bioscience), anti-p27 (mouse, 1∶2000, BD Transduction Laboratories), and anti-p21 (mouse, 1∶50, Santa Cruz Biotechnology). Horseradish peroxidase (HRP) conjugated secondary antibodies (BioRad) were used to detect primary antibodies and 3, 3′-diaminobenzidine (Sigma) or Nova Red (Vector Laboratories) were used as the chromogenic substrates. Counterstain was performed with hematoxylin.

### Immunohistochemistry quantification

Three independent fields of anterior prostate at 60× were observed for 8-OHdG immunohistochemical staining in one year old *Nkx3.1^+/+^* and *Nkx3.1^−/−^* mice and for BrdU, pHH3, and/or activated caspase 3 staining in the *Nkx3.1^+/+^* and *Nkx3.1^−/−^* vehicle and NAC-treated mice. Number of total cells and cells staining positive for each of the markers were recorded and data was reported as percent cells positive for the marker. In all cases, at least 500 total cells were counted per mouse.

### Statistical analysis

Statistical analysis for immunohistochemisrty, qRT-PCR, and fluorescence intensity image data was performed using two tailed Student's t-Test, with two samples of unequal variance. All results are presented as mean ± Standard Deviation. P values≤0.05 are considered significant.

### Microarray and Genome Set Enrichment Analysis (GSEA)

Total RNA from four vehicle and four NAC-treated *Nkx3.1^−/−^* mice was extracted from snap-frozen mouse anterior prostate tissue according to the Trizol® manufacturer's protocol. RNA was treated with RQ1 Rnase-free DNAse (Promega) according to manufacturer's protocol, followed by purification using the RNA Clean Up protocol from the RNeasy Mini Kit (Qiagen). RNA was processed and microarray analysis was performed by the Vanderbilt Genome Sciences Resource Core. Briefly, RNA was quantified using the Qubit RNA assay and RNA quality was assessed with the Agilent Bioanalyzer. cDNA was generated using the Ambion® WT Expression Kit. After fragmentation, the cDNA was labelled and hybridized to Affymetrix Mouse Gene 1.0 ST arrays. Arrays were scanned with Affymetrix Gene Chip Scanner [version 3.2.2]. CEL files were imported to R [version 2.15.1] for quality control and pre-processing. Arrays for three vehicle and four NAC-treated mice passed quality control. Using the Affy package [version 1.34.0] [42], raw intensity scores for probes were normalized by quantiles, background corrected with RMA [43], and summarized by median polish using PM-only probes. The C2 (curated) gene sets of MSigDB [version 3.0] were queried using GSEA [version 2.07] [44] to test for differences between vehicle and NAC-treated prostates. Relationships between functional terms were visualized in Cytoscape [version 2.8.3] [45] with the Enrichment Map package [version 1.2] [46]. All microarray and GSEA analysis was performed on a node running Debian Linux [version 6.0.5].

## Results

### Nkx3.1 directly regulates antioxidant and pro-oxidant genes in the prostate

**Figure 1:**
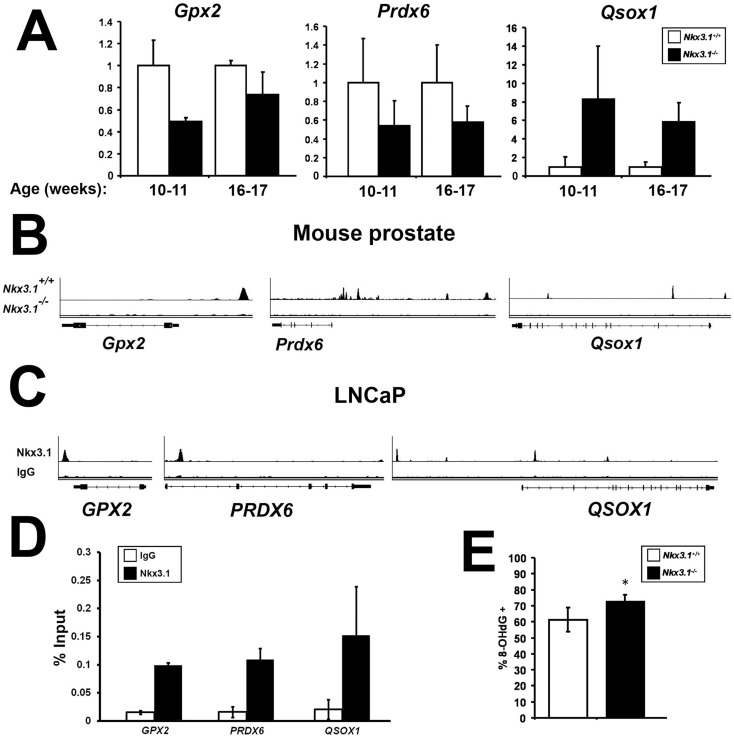
*Nkx3.1^−/−^* mouse prostate shows dysregulation of oxidative stress genes and increased oxidative stress levels. (A) Quantitative reverse transcriptase-PCR analysis of RNA from 10–11-week and 16–17-week-old *Nkx3.1^+/+^* and *Nkx3.1^−/−^* mouse anterior prostate for the expression of *Gpx2*, *Prdx6*, and *Qsox1*. Expression levels are relative to 18s rRNA. (10–11 weeks: n = 4 *Nkx3.1^+/+^*, n = 2 *Nkx3.1^−/−^*; 16–17 weeks: n = 3 *Nkx3.1^+/+^*, n = 5 *Nkx3.1^−/−^*) (B) ChIP-seq screen shots from Integrative Genomics Viewer (IGV) displays direct binding of Nkx3.1 to the gene loci of *Gpx2*, *Prdx6* and *Qsox1* in mouse prostate, (C) and to *GPX2*, *PRDX6* and *QSOX1* in the human prostate cancer cell line LNCaP. (D) ChIP-qPCR analysis for Nkx3.1 binding sites in *GPX2*, *PRDX6*, and *QSOX1*. Results are presented for each binding site primer set with anti-NKX3.1 antibody and IgG control. Immunoprecipitated DNA was normalized to 1% input. (E) Percent positive stained anterior prostate epithelial cells from immunohistochemical staining for 8-OHdG in one-year-old *Nkx3.1^+/+^* and *Nkx3.1^−/−^* anterior prostate. (n = 5 in each group) Student's t-Test * = p≤0.05.

Previous gene expression analyses studies have revealed mis-expression of antioxidant and pro-oxidant genes in the *Nkx3.1* null mouse prostate, including Glutathione peroxidase (*Gpx2*), Peroxiredoxin 6 (*Prdx6*), and quiescin Q6 sulfhydryl oxidase 1 (*Qsox1* or *Qscn6*) [26], [39], [47]. We performed qRT-PCR analysis on anterior prostates to confirm these gene expression changes. Expression of the antioxidant genes Gpx2 and Prdx6 was decreased in 10–11-week-old and 16–17-week-old *Nkx3.1^−/−^* mice, while expression of the pro-oxidant gene Qsox1 was elevated in these mice (Figure 1A). Examination of chromatin immunoprecipitation coupled to massively parallel sequencing (ChIP-seq) analysis for Nkx3.1 in mouse prostate [47] and the human prostate cancer cell line LNCaP (PDA, ML and SAA, manuscript in preparation) performed by our laboratory revealed binding sites for Nkx3.1 in both human and mouse tissue at all three genes (Figure 1B, 1C). Therefore, Gpx2, Prdx6 and Qsox1 are direct target genes of the Nkx3.1 transcription factor.

### *Nkx3.1^−/−^* mouse prostate displays increased oxidative stress

The most common oxidative DNA base lesion, 8-OHdG, is commonly used as a marker of persistent oxidative stress [48]. Immunohistochemical staining of one-year-old mouse anterior prostate showed significantly increased staining in *Nkx3.1^−/−^* mice (Figure 1D). These results confirm earlier findings of increased oxidative DNA damage in the prostates of independently generated *Nkx3.1^−/−^* mice [39].

### NAC treatment of *Nkx3.1^−/−^* mice does not inhibit hyperplastic prostate phenotype

**Figure 2:**
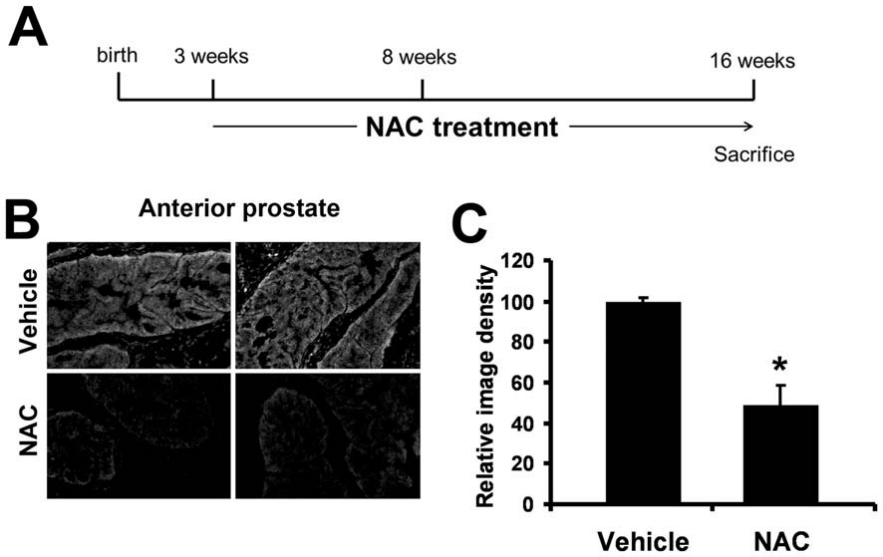
Antioxidant treatment of *Nkx3.1^−/−^* mice decreases prostatic ROS. (A) *Nkx3.1^−/−^* mice were treated with 5 mM N-acetylcysteine (NAC) *ad lib* in their drinking water postweaning for 13 weeks. Mice were sacrificed for analysis at the end of treatment (16 weeks of age). (B) Dihydroethidium (DHE) staining of frozen anterior prostate from *Nkx3.1^−/−^* vehicle or NAC-treated mice. (C) Quantification of DHE staining density. (n = 3 in each group) Student's t-Test * = p≤0.05.

To determine if increased oxidative stress plays a causative role in the hyperplasia and dysplasia observed in the *Nkx3.1^−/−^* mouse prostate, we treated *Nkx3.1^−/−^* mice with 5 mM NAC in their drinking water from 3 weeks of age until mice were sacrificed at 16 weeks of age (Figure 2A). The 5 mM NAC concentration was chosen to achieve a dosage of approximately 125 mg/kg/day for 13 weeks, a dosage and treatment duration shown to inhibit plasma ROS, decrease oxidative DNA and protein lesions in the prostate, and decrease the incidence of prostate anterior lobe hyperplasia in the Transgenic Adenocarcinoma Mouse Prostate (TRAMP) model [7], [8]. Examination of water intake and weight data revealed that the achieved dosage for the *Nkx3.1^−/−^* mice was approximately 140 mg/kg/day. The 13 week NAC treatment decreased ROS levels in the anterior prostate as shown by decreased staining for superoxide using the fluorescent dye dihydroethidium (DHE) (Figure 2B, 2C).

**Figure 3:**
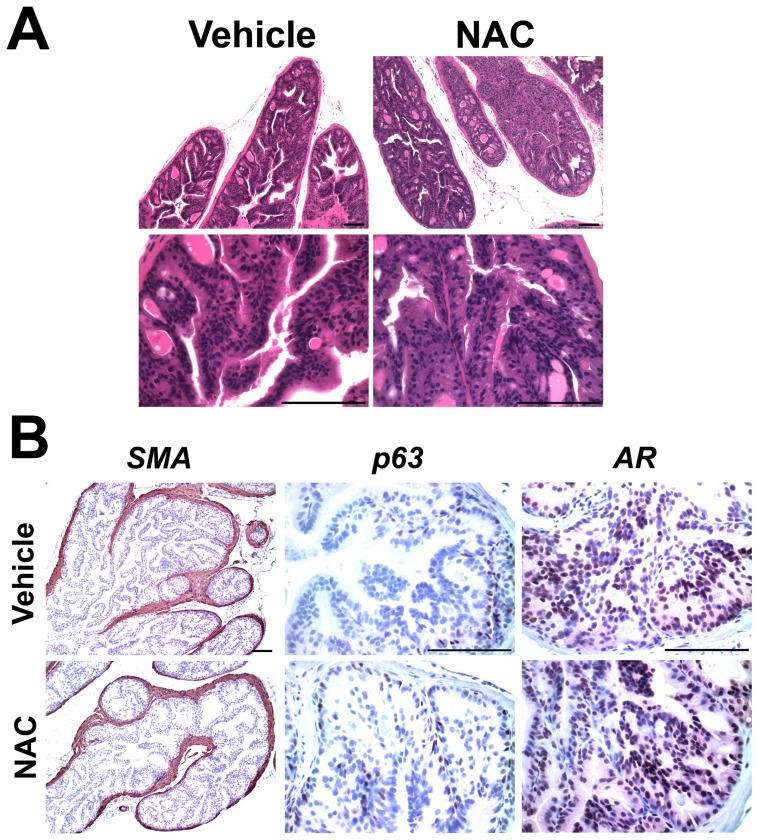
NAC treatment does not alter prostate histology in *Nkx3.1^−/−^* mice. (A) Hematoxalin and eosin stained sections of *Nkx3.1^−/−^* anterior prostate do not display significant histological changes with NAC treatment. (B) Immunohistochemical staining of anterior prostate for smooth muscle actin (SMA), p63, and androgen receptor (AR) do not have significant changes in staining pattern. Scale bar = 0.1 mm.

**Figure 4:**
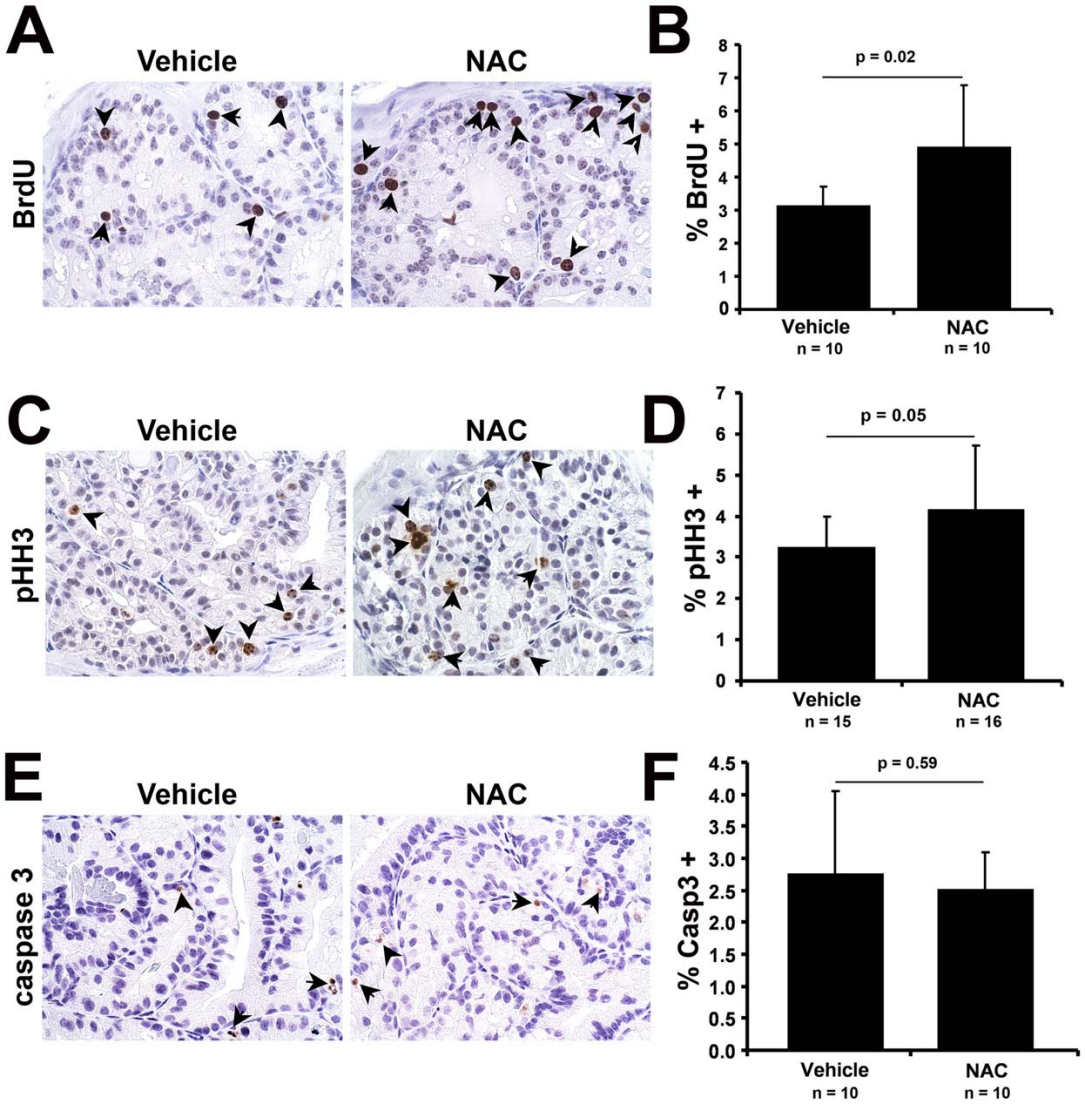
NAC treatment promotes epithelial proliferation in the *Nkx3.1^−/−^* prostate. (A), (C), (E) Representative images from immunohistochemical staining of *Nkx3.1^−/−^* vehicle and NAC-treated anterior prostate with antibodies specific to BrdU (A), pHH3 (C), and activated caspase-3 (E). (B), (D), (F) Quantification of immunohistochemical stains. p values for a Student's t-Test are shown.

Histological analysis of *Nkx3.1^−/−^* anterior prostate, the prostatic lobe which displays the severest *Nkx3.1^−/−^* phenotype, showed that the NAC treatment did not reverse the *Nkx3.1^−/−^* phenotype. Observation of 23 control and 24 NAC-treated *Nkx3.1^−/−^* prostates revealed that the NAC-treated prostates did not have less hyperplasia or dysplasia than the control prostates (Figure 3A). Immunohistochemical staining for smooth muscle actin was unchanged between treated and untreated mice, suggesting the prostate epithelial cells did not alter gland structure or invade the stromal compartment (Figure 4B). Immunostaining for p63 (basal cell marker) and androgen receptor (AR) remained unchanged with treatment, showing no major histological alterations of the prostate epithelium after NAC treatment (Figure 3B).

### NAC treatment of *Nkx3.1^−/−^* mouse prostate promotes increased proliferation

To assess cell proliferation in the prostate after NAC treatment, mice were injected with BrdU three hours prior to sacrifice to label cells undergoing DNA synthesis, indicating the proportion of cells progressing though the cell cycle. Surprisingly, the percentage of anterior prostate epithelial cells staining positive for BrdU was increased by 60% in the NAC-treated *Nkx3.1^−/−^* mice (p = 0.02, n = 10 in each group, Figures 4A, 4B). Staining for the mitotic cell marker pHH3 was also increased by 30% in the NAC-treated animals (p = 0.05, n = 15 vehicle, n = 16 NAC, Figures 4C, 4D). However, activated caspase-3 staining revealed that apoptosis was unchanged with NAC treatment (p = 0.59, n = 10 in each group, Figures 4E, 4F). The observed increase in proliferation without a concurrent decrease in apoptosis suggests NAC treatment increases prostate epithelial cell numbers in the *Nkx3.1^−/−^* prostate.

### NAC treatment of *Nkx3.1^+/+^* mouse prostate does not affect proliferation

**Figure 5:**
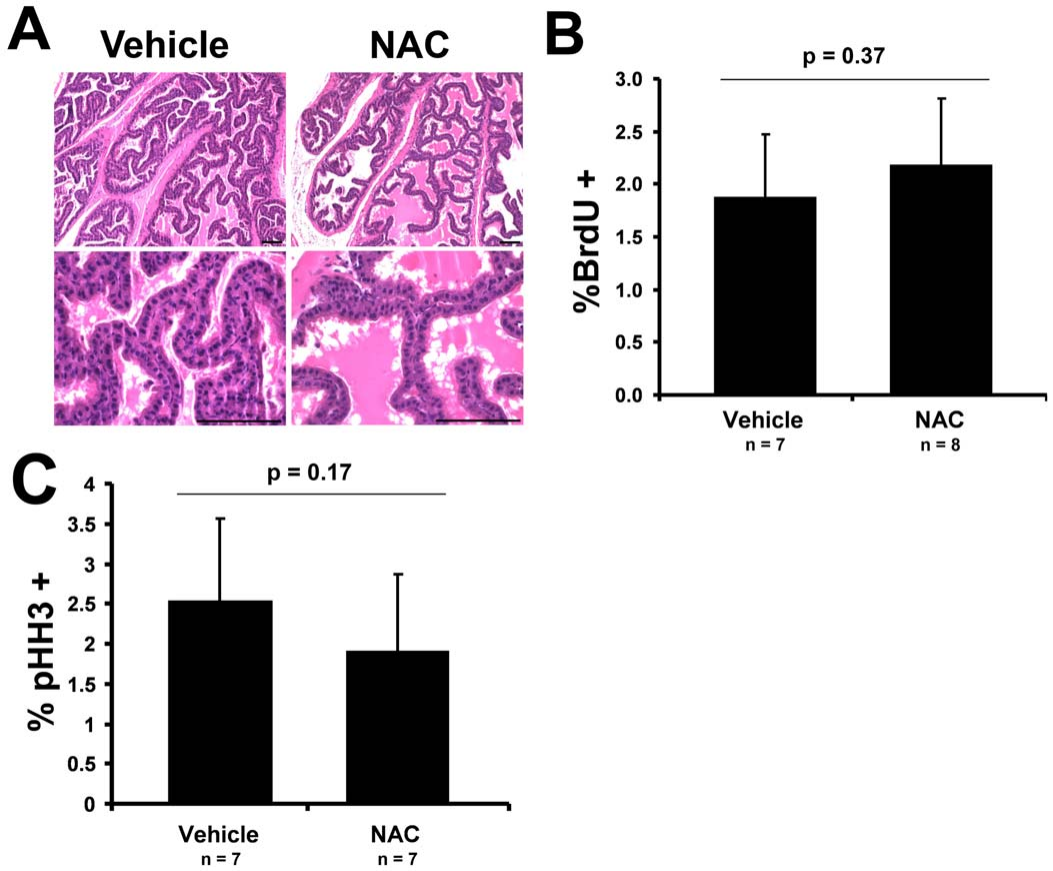
NAC treatment does not alter epithelial proliferation in the *Nkx3.1^+/+^* prostate. (A) H&E sections of *Nkx3.1^+/+^* vehicle and NAC-treated anterior prostate show no change in histology. Scale bar = 0.1 mm. (B) Quantification of BrdU immunohistochemical staining in *Nkx3.1^+/+^* vehicle and NAC-treated anterior prostate. (C) Quantification of pHH3 immunohistochemical staining in *Nkx3.1^+/+^* vehicle and NAC-treated anterior prostate. p value for a Student's t-Test is shown.

To determine if NAC treatment affects prostate epithelial cell proliferation in the absence of Nkx3.1-loss and elevated oxidative stress, we treated *Nkx3.1^+/+^* mice with NAC in the same manner as was used for the *Nkx3.1^−/−^* mice. The dosage achieved in the *Nkx3.1^+/+^* mice was comparable to the *Nkx3.1^−/−^* mice at approximately 160 mg/kg/day. The NAC treatment did not alter overall prostate histology in the *Nkx3.1^+/+^* mice (Figure 5A). BrdU and pHH3 immunohistochemical analyses showed that NAC treatment did not alter the proliferation index of the *Nkx3.1^+/+^* anterior prostate (Figure 5B, 5C).

### NAC treatment of the *Nkx3.1^−/−^* mouse prostate promotes expression of a pro-proliferative gene signature

**Figure 6:**
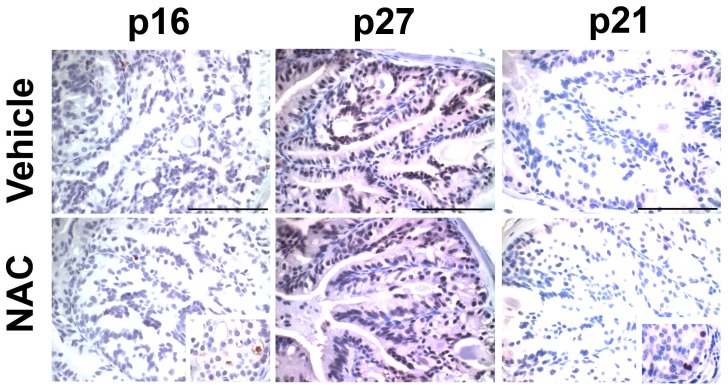
NAC treatment of the *Nkx3.1^−/−^* prostate does not alter expression of well-established senescence and quiescence markers. Immunohistochemical staining of *Nkx3.1^−/−^* vehicle and NAC-treated anterior prostate for p16, p27, and p21. *p16 inset*: positive control for p16 staining from *PbCre4; Pten^f/f^* prostate [73]. *p21 inset*: positive control for p21 staining from *PbCre4; Pten^f/f^; p53^f/+^* prostate [74]. Scale bar = 0.1 mm.

ROS have been shown to induce senescence and quiescence in human and mouse models of disease [49]. Because quenching of prostatic ROS with NAC increased epithelial cell proliferation, we hypothesized that oxidative stress in the *Nkx3.1*-null prostate induces cell cycle arrest. We performed immunohistochemical staining for well-defined markers of senescence (p16, p21) and quiescence (p27) in *Nkx3.1^−/−^* vehicle and NAC-treated prostates. Expression of these markers remains unchanged with NAC treatment (Figure 6).

In order to analyze global gene expression changes associated with NAC treatment of the *Nkx3.1^−/−^* prostate, we performed Affymetrix microarray analysis on total RNA extracted from three *Nkx3.1^−/−^* vehicle and four *Nkx3.1^−/−^* NAC-treated anterior prostates. Genome Set Enrichment Analysis (GSEA) [44],[50] is used to determine if the expression of *a priori* defined gene sets, relating to biological pathways or experimental conditions, is significantly altered in the experimental tissue of interest. GSEA allows for detection of modest gene expression changes of many genes in one pathway that as a group may have a functional biological effect. The GSEA Molcular Signatures Database (MSigDB) collections consist of sets of human genes. We compared our mouse gene expression data to the human gene sets using the human genes orthologous to the mouse genes. Using the C2 (curated) gene sets collection, we identified many gene sets that were significantly enriched or depleted in NAC-treated *Nkx3.1^−/−^* prostates, including several that are associated with proliferation control and quiescence (Tables S1 and S2).

**Figure 7:**
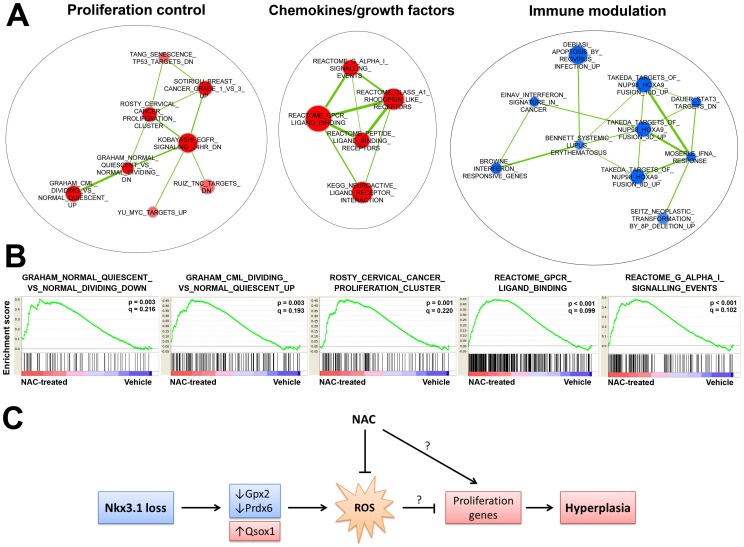
NAC treatment promotes proliferation of a pro-proliferative gene expression signature in *Nkx3.1^−/−^* prostate. (A) Enrichment Map [46] analysis for Genome Set Enrichment Analysis (GSEA) C2 (curated) gene set data obtained from vehicle and NAC-treated *Nkx3.1^−/−^* anterior prostate. Map displays the related gene networks containing ≥5 gene sets with a false discovery rate (FDR) q value <0.25. Node size corresponds to gene set size. Hue designates which manner in which the gene sets are altered (red = enriched in NAC-treatment, blue = depleted in NAC-treatment). Color intensity represents significance by enrichment p value. Line thickness connecting the gene set nodes represents the degree of gene overlap between the two sets. (B) GSEA Enrichment plots [44], [50] for selected gene sets from the “proliferation control” network and the “chemokines/growth factors” network. Nominal p value (statistical significance of the enrichment) and the FDR are presented. (C) Potential model for Nkx3.1-loss associated ROS and NAC treatment in prostate tumor initiation.

To obtain a broader picture of the relationships between the significantly altered gene sets in NAC-treated *Nkx3.1^−/−^* prostates, we performed Enrichment Map analysis [46]. This is a method for GSEA interpretation and visualization which constructs networks from gene sets (nodes) containing overlapping genes. Analysis of identified networks using Enrichment Map can yield important information about the broad biological processes altered in a treatment group. Enrichment Map results for all networks containing ≥5 nodes are presented in Figure 7A. The first network we term “proliferation control” and consists of 7 nodes. One of these upregulated “proliferation control” gene sets (GRAHAM_NORMAL_QUIESCENT_VS_NORMAL_DIVIDING_DN) is a gene set consisting of transcripts that are downregulated during quiescence of hemopoetic stem cells (HSCs) and another is a set upregulated in dividing leukemia stem cells compared to quiescent HSCs (GRAHAM_CML_DIVIDING_VS_NORMAL_QUIESCENT_UP) (Figure 7B,[51]). Another upregulated “proliferation control” gene set is ROSTY_CERVICAL_CANCER_PROLIFERATION_CLUSTER, consisting of genes controlling cell division and proliferation and associated with an increased severity and early relapse in cervical cancer (Figure 7B, [52]). Enrichment of this network in the NAC-treated prostate serves as further quantitative evidence of increased proliferation in *Nkx3.1^−/−^* prostate upon NAC treatment. Another network upregulated in the NAC-treated *Nkx3.1^−/−^* prostates contains gene sets comprised in a large part by chemokine/growth factor genes such as REACTOME_G_ALPHA_I_SIGNALLING_EVENTS (Figure 7A, 7B). A network consisting of sets involved in immune regulation was depleted in NAC-treated *Nkx3.1^−/−^* prostates (Figure 7A).

The ‘leading edge’ is the subset of genes within a specific MSigDB gene set which drives the observed association in GSEA. Analysis of the leading edge genes may help to determine which changes in gene expression are responsible for a given phenotype. Leading edge genes from the “proliferation control” network (Table 1) include many classic pro-proliferative genes such *Ccna2* (*CCNA2* in human), *Cdc6*, *Tk1*, and *Gmnn*. Leading edge genes in the “chemokines/growth factors” network (Table 2) include many involved in pathways that have proven links to prostate cancer, including chemokines/chemokine receptors (*Ccl2, Cxcl5, Cxcr1, Cxcr2*) [53],[54], the endothelin axis (*Ednrb, Ednra*) [55], and neuropeptides (*Npy, Npy1r, Npy5r, Pyy*) [56].

**Table 1:** Leading edge genes from a sample of “proliferation control” gene sets with significant enrichment.

Gene set name	Leading edge genes
**GRAHAM_NORMAL_QUIESCENT_ VS_NORMAL_DIVIDING_DN**	CD36, TK1, CPA3, RACGAP1, DLGAP5, CDC6, PRC1, COTL1, DTL, BUB1, MCM10, CDC20, CCNB2, RRM2, MCM6, MELK, NDC80, CCNA2, CENPM, GMNN, RAD51AP1
**GRAHAM_CML_DIVIDING_ VS_NORMAL_QUIESCENT_UP**	CD36, TUBB6, CCL2, SERPINB2, XIST, PF4, TK1, CPA3, HGF, RACGAP1, FAM38B, DLGAP5, CDC6, MPO, PRC1, COTL1, BUB1, MCM10, CDC20, CCNB2, PBK, RRM2, PPBP, UBE2S, CDC7, TPX2, CLEC11A, NEK2, MICAL2, MELK, NDC80, ASPM, KPNA2, HMMR, CCNA2, CENPM, GMNN, RAD51AP1, BRCA1, ECT2, PMP22, AURKA, CSTA, ESPL1, ACOT7, ELOVL6
**ROSTY_CERVICAL_CANCER_ PROLIFERATION CLUSTER**	TK1, SHCBP1, NETO2, RACGAP1, DLGAP5, HN1, PLK1, CDC6, MKI67, PRC1, CDCA3, DTL, BUB1, ASF1B, E2F1, MCM10, CDC20, CCNB2, PBK, RRM2, CDCA8, UBE2S, DBF4, TPX2, NEK2, MELK, NDC80, ASPM, KPNA2, CELSR3, HMMR, CCNA2, CENPM, GMNN, RAD51AP1, BRCA1, ECT2, AURKA, ESPL1, HMGA1, AURKB, NCAPH, TACC3, TTK, E2F8, LRP8, LMNB1

**Table 2:** Leading edge genes from a sample of “chemokines/growth factors” gene sets with significant enrichment.

Gene set name	Leading edge genes
**REACTOME_GPCR_ LIGAND_BINDING**	EDNRB, CXCR2, CCL7, CCL2, CXCL13, FFAR1, PF4, NPY, NPY1R, OPN4, C3, HTR5A, ADORA2B, GRM3, HEBP1, PROK2, CCL3, S1PR3, CCL11, NPS, C5AR1, CNR1, AVPR1B, VIP, SSTR1, FPR1, ANXA1, CALCRL, OPRM1, P2RY13, WNT2B, PDYN, UTS2, F2, TSHR, UTS2R, S1PR2, CCL4, GNG3, TAC1, CXCL11, APLN, GNB3, HRH3, DARC, HTR1A, AVPR1A, ADORA1, ADORA3, DRD5, TAS1R2, TACR3, FSHB, NPY5R, CCR3, CCL22, PPBP, RHO, HTR1D, HTR4, HCRT, BDKRB2, C3AR1, MC4R, ADM2, APLNR, CXCR3, TAS1R1, SSTR2, WNT6, OPRL1, GRM5, PROKR2, ADRA1D, LPAR4, OPRK1, FZD4, CHRM5, NPSR1, TAAR1, GPBAR1, MC2R, FFAR2, WNT4, WNT8A, HTR6, CCL17, CXCR5, SCT, ADCYAP1, ADRB3, LPAR1, TSHB, SSTR3, SSTR4, OPRD1, GHRHR, TRH, HRH4, PYY, CCL25, CCR10, OPN5, GALR2, QRFPR, HCRTR2, ADRA2C, CXCR1, GPR17, AGT, PPYR1, FZD10, CALCB, KISS1R, CASR, CCR7, EDNRA, HTR1B, CRHR2, MTNR1B, P2RY2, BDKRB1, HRH1, PRLH, CCR1, TRHR, OXT, P2RY4, GIPR, CXCL5
**REACTOME_G_ALPHA_ I_SIGNALLING_EVENTS**	CXCR2, CXCL13, PF4, NPY, NPY1R, C3, HTR5A, HEBP1, ADCY2, S1PR3, C5AR1, CNR1, ADCY4, SSTR1, FPR1, ANXA1, OPRM1, P2RY13, PDYN, S1PR2, GNG3, CXCL11, APLN, GNB3, HRH3, HTR1A, ADORA1, ADORA3, ADCY10, NPY5R, CCR3, PPBP, RHO, HTR1D, BDKRB2, C3AR1, APLNR, CXCR3, SSTR2, OPRL1, OPRK1, CXCR5, LPAR1, SSTR3, SSTR4, OPRD1, HRH4, PYY, CCL25, CCR10, OPN5, GALR2, ADRA2C, CXCR1, GPR17, AGT, PPYR1, CASR, CCR7, HTR1B, MTNR1B, BDKRB1, ADCY8, CCR1, GNAT1, P2RY4, CXCL5

## Discussion

Our study has displayed novel evidence of prostate tumor promotion by antioxidant treatment. Using *Nkx3.1*-null mice, we have modeled antioxidant chemoprevention in the early stages of prostate tumorigenesis and shown an increase in prostate epithelial proliferation upon NAC treatment. These results suggest that ROS can be anti-tumorigenic in the early stages of prostate cancer and that antioxidant chemoprevention may be ineffective or harmful in many circumstances.

In this report we have confirmed that *Nkx3.1^−/−^* mice display increased prostatic oxidative stress. The hyperproliferative state of the *Nkx3.1^−/−^* prostate may promote increased oxidative stress through one of many indirect mechanisms. However, we have shown that the oxidative stress regulatory genes *Gpx2*, *Prdx6*, and *Qsox1* are dysregulated in the mutant mice and are shown to be direct targets of the the Nkx3.1 transcription factor in both the mouse and human prostate. Therefore, we propose that loss of Nkx3.1 expression may directly affect oxidative stress maintenance through dysregulation of these target genes.

To determine if elevated oxidative stress is a causative mechanism for the hyperplasia observed in the *Nkx3.1^−/−^* prostate, we treated *Nkx3.1^−/−^* mice with the antioxidant NAC. NAC is a precursor for the most prevalent antioxidant molecule in cells, glutathione (GSH). NAC has been safely used for many years in mice and humans and has been shown in previous studies to increase GSH concentration, decrease oxidative stress, and have beneficial clinical effects [57], [58]. While NAC treatment did decrease ROS levels in the *Nkx3.1^−/−^* prostate, it did not alter the hyperplastic phenotype. Upon immunohistochemical staining with BrdU and pHH3, we observed that NAC treatment promoted proliferation in the *Nkx3.1^−/−^* prostate. Surprisingly, rather than inhibit the hyperplastic phenotype, NAC treatment promotes hyperplasia in the *Nkx3.1^−/−^* prostate. In the *Nkx3.1^+/+^* prostate, NAC treatment did not increase proliferation, suggesting that the mechanism by which NAC increases proliferation in the *Nkx3.1^−/−^* prostate is related to elevated oxidative stress.

Because we observed increased proliferation in the NAC-treated *Nkx3.1^−/−^* prostate, we hypothesized that elevated ROS in the *Nkx3.1^−/−^* prostate is activating an anti-proliferative pathway or inhibiting a pro-proliferative pathway, reducing the proliferative potential of the prostate epithelial cells. Antioxidant treatment would suppress this ROS-mediated effect, allowing for the epithelial cells to proliferate more. To first test this hypothesis, we performed immunoshistochemical analysis of well-established senescence and quiescence markers. This did not reveal any changes with NAC treatment of the *Nkx3.1^−/−^* prostate. To further investigate the possible mechanism behind the increased proliferation upon NAC treatment, we performed global gene expression analysis on vehicle and NAC-treated *Nkx3.1^−/−^* prostate. Analysis of the gene expression data with GSEA and Enrichment Map revealed a significant enrichment in expression of gene sets involved in proliferation control and chemokine/growth factor function. Increased expression of this pro-proliferative gene signature, consisting of classic proliferation genes (i.e. cyclins) and chemokines/growth factors, many of which have been implicated in prostate cancer [53], [54], [55], [56], may explain the increase in proliferation seen upon NAC treatment of the *Nkx3.1^−/−^* prostate.

Based upon our findings, we propose a potential model for Nkx3.1-loss associated ROS and NAC treatment in prostate tumor initiation (Figure 7C). Loss of Nkx3.1 expression in the prostate causes dysregulation of antioxidant and pro-oxidant direct target genes, resulting in elevated ROS in the hyperplastic *Nkx3.1^−/−^* prostate. These ROS may actually limit proliferation in the *Nkx3.1^−/−^* prostate by inhibiting expression of pro-proliferative genes. ROS have been shown to induce cell cycle arrest or decrease proliferation in several models of non-cancerous and cancerous cells [59], [60], [61], [62], and, in some of these cases, antioxidant treatment has been explicitly shown to reverse these ROS-induced effects. Thus, NAC may be increasing proliferation of the *Nkx3.1*-null prostate by decreasing ROS-mediated inhibition of pro-proliferative genes. Alternatively, NAC may promote proliferation by an alternative mechanism independent of prostatic ROS inhibition.

Results from this study emphasize the need for a deeper understanding of the role reactive oxygen species play in prostate tumor progression. The effect of ROS on cells is not always pro-tumorigenic. The level of ROS present in a tissue can influence the effect seen, with high levels of ROS promoting senescence or cell death, but lower levels promoting DNA mutations or activating pro-proliferative signaling. The cell type with which ROS interacts also determines its effect. In a normal cell, a certain level of ROS may kill the cell or cause a cell to undergo cell cycle arrest, while in a cancer cell the same level of ROS may promote proliferation and invasion.

Despite the ability of antioxidants to inhibit cancer in several mouse models, we have shown that the antioxidant NAC promotes proliferation in the *Nkx3.1^−/−^* prostate. We propose that the *Nkx3.1^−/−^* mouse is a good model for antioxidant chemoprevention, exhibiting early lesions similar to those of cancer-naïve men in whom clinicians desire to prevent malignant disease. Indeed, antioxidants may function to inhibit tumor progression at later stages, effectively treating cancer in some settings. However, at early stages, ROS may actually slow or prevent tumor progression from occurring [63], [64]. In addition, different antioxidant compounds may affect the prostate in unique ways. Alternatively, ROS may have different effects on prostate tumor progression based on the genetic lesions or gene expression changes present.

The recent alarming results from the SELECT trial, in which “antioxidant chemoprevention” increased prostate cancer risk, can be informed by our study. While the proliferation upon NAC treatment is not increased to an extremely large degree, it is nevertheless a significant increase and could become more pronounced with long term treatment. NAC was not the specific antioxidant used in the SELECT trial; however, the results can yield important information due to the fact that NAC should decrease the overall oxidative state and possibly reflect possible results seen by other antioxidants. In fact, a recently published study using selenium and vitamin E in a rat model of prostate tumorigenesis showed a similar finding, that vitamin E treatment showed a marginally significant increase in prostate tumor formation [65].

In the setting of certain genetic lesions or expression changes, such as Nkx3.1-loss, depleting ROS may actually allow cells to escape a ROS-mediated inhibition of proliferation, increasing the chance of transformation of the prostate epithelium. The increased prostate cancer risk in the SELECT study population may indeed be driven by a subset of participants with an inherited polymorphism in Nkx3.1 (rs11781886) that is associated with increased prostate cancer risk [66]. Depletion of ROS by vitamin E may have modified the risk from the levels normally associated with the polymorphism, producing the surprising detrimental effect with vitamin E chemoprevention. Oxidative stress and antioxidant levels have been shown in previous studies to modify cancer risk associated with inherited polymorphisms [67], [68], [69], [70], [71]. Studies are ongoing using the SELECT biorepository to determine if antioxidant treatment increased the prostate cancer risk associated with the functional Nkx3.1 variant (rs11781886) [72].

Our report provides valuable insight into the inconsistent results among preclinical and clinical studies on the efficacy of prostate cancer antioxidant chemoprevention [6]. We suggest that investigation of prostate cancer chemoprevention specifically in physiologically relevant models, with analysis of the complexities of specific gene expression changes, is critically needed if clinically applicable results are desired. Caution should be taken when using antioxidants for prostate cancer prevention, because the effect which they have, beneficial or harmful, may lie in the makeup of the prostate gland of each unique individual.

## Supporting Information

Table S1 Top gene sets enriched in NAC-treated *Nkx3.1^−/−^* anterior prostate. GSEA analysis results for top gene sets enriched in NAC-treated *Nkx3.1^−/−^* anterior prostate as ranked by Normalized Enrichment Score. Gene set size, Enrichment Score, Normalized Enrichment Score, Nominal p-value, and FDR q-value are provided. Enriched gene sets with a FDR q-value<0.25 are provided (99 gene sets).
(XLSX)

Table S2 Top gene sets depleted in NAC-treated *Nkx3.1^−/−^* anterior prostate. GSEA analysis results for top gene sets depleted in NAC-treated *Nkx3.1^−/−^* anterior prostate as ranked by Normalized Enrichment Score. Gene set size, Enrichment Score, Normalized Enrichment Score, Nominal p-value, and FDR q-value are provided. Enriched gene sets with a FDR q-value<0.25 are provided (29 gene sets).
(XLSX)
